# Povidone-Iodine Irrigation of Subcutaneous Tissues May Decrease Surgical Site Infections in Elective Colorectal Operations: A Systematic Review

**DOI:** 10.4021/gr319e

**Published:** 2011-05-20

**Authors:** Richdeep S Gill, David P Al-Adra, Sandy Campbell, David W Olson, Brian H Rowe

**Affiliations:** a Department of Surgery, University of Alberta, Edmonton, Alberta, Canada; b Library Services, University of Alberta, Edmonton, Alberta, Canada; c Department of Emergency Medicine and School of Public Health, University of Alberta, Edmonton, Alberta, Canada

**Keywords:** Povidone-Iodine, Colorectal operation, Surgical site infection

## Abstract

**Background:**

Postoperative wound infection is the most common complication following abdominal surgery and leads to delayed wound healing, prolonged hospital length of stay (LOS), and causes morbidity. Povidone-Iodine (PVI) is a broad-spectrum anti-septic and disinfectant solution, and can be used intra-operatively to irrigate subcutaneous tissues prior to abdominal skin closure. We systematically reviewed the literature regarding the efficacy of intra-operative PVI irrigation of subcutaneous tissues following elective colorectal surgery.

**Methods:**

A comprehensive search of electronic databases and various grey literature sources was completed. Unpublished and non-English-language results were included. All clinical controlled trials involving PVI solution in adult colorectal surgery were included. Two independent reviewers assessed the studies for relevance, inclusion, methodological quality and extracted data from the full versions of the manuscripts. Disagreements were resolved by re-extraction or third party adjudication. Data for dichotomous outcomes are reported as relative risks (RR) with 95% confidence intervals (CI). For continuous data, mean differences (MD) are reported with 95% CIs.

**Results:**

Five randomized controlled trials (RCTs) involving 205 patients comparing PVI solution or spray to a control group following abdominal fascial closure in elective colorectal or clean-contaminated operations were identified. Pooled results demonstrated a reduction in surgical site infection for patients treated with PVI (RR = 1.97; 95% CI: 1.22 to 3.17) compared to controls.

**Conclusions:**

Irrigation of subcutaneous tissues with PVI following abdominal fascial closure is associated with a reduced incidence of surgical site infection. Due to the small number of included trials and patients, additional robust randomized trials are needed.

## Introduction

### Description of the condition

Postoperative wound infection is the most common complication following abdominal operations [1]. Surgical site infections (SSIs) account for 40% of nosocomial infections among surgical patients [2]. The risk of wound infection is increased following a clean-contaminated operation such as elective colorectal surgery. It is estimated that surgical site infection rates for clean-contaminated surgery are approximately 5 - 15%. Despite improvement in sterile surgical technique, preoperative antibiotics and awareness of surgical site infections, wound infection rates have not dramatically changed. Wound infection at the surgical site delays wound healing, prolongs hospital length of stay (LOS) [3] and may lead to wound dehiscence. Severe consequence of SSIs such as longer hospitalization and greater mortality are more likely in older patients [4].

### Description of the intervention

Povidone-Iodine (PVI) is a broad-spectrum anti-septic and disinfectant solution [5], which is reportedly capable of killing bacteria on contact. PVI functions by releasing free iodine, which binds with protein [6]. Once bound, the iodine is carried across the cell membrane into the cytoplasm of cells by polyninylpyrrolidone [7]. The free iodine has a microbicidal effect within 15 seconds [8]. PVI is used both as a pre-operative skin cleanser and intra-operatively. In the intra-operative approach, the subcutaneous abdominal tissues are irrigated with PVI to cleanse the tissue of pathogens that may lead to surgical site infections. PVI can be applied either as a solution or spray at varying concentrations.

### How the intervention might work

PVI solution applied to the abdominal subcutaneous tissues prior to skin closure may kill the enteric organisms that can contaminate the abdominal incision during surgery. In addition, PVI can release free iodine, which may sterilize the skin surrounding the incision, and prevent skin flora from causing a wound infection.

### Why it is important to do this review

Nosocomial surgical site infections are not benign events. Patients who develop SSIs are five times more likely to be readmitted to hospital, 60% more likely to spend time to intensive care unit and twice as likely to die as patients without SSIs [9]. The effectiveness of PVI in reducing wound infection rates has been studied; however, agreement on its effectiveness in preventing SSI remains controversial.

### Objectives

To systematically review the literature regarding the effectiveness of intra-operative Povidone-Iodine irrigation of subcutaneous tissues in preventing surgical site infection, following elective colorectal surgery.

## Methods

### Criteria for considering studies for this review

#### Types of studies

Randomized or quasi-randomized controlled trials. Clinically controlled trials (CCTs).

#### Types of participants

The target population consists of adult (> 18 years old) male or female patients undergoing elective colorectal operations. Elective colorectal operations performed via an abdominal incision for cancer, diverticulosis and other pathologies were included. We considered clean-contaminated abdominal surgery to represent this population. When encountered, mixed populations were considered for inclusion if more than 80% of cases involving an abdomen incision were reported.

#### Types of interventions

The intervention under study was PVI for subcutaneous irrigation of the abdominal incision. We considered comparisons between different concentrations of PVI for irrigation, or comparisons of PVI to normal saline or sterile water.

#### Types of outcome measures

1) Primary outcomes: the primary outcome was surgical site infection rates.

2) Secondary outcomes: (1) Hospital LOS; (2) Re-hospitalization; (3) Dehiscence; (4) Re-exploration; (5) Sepsis; (6) Death; (7) Health economic outcomes.

### Search methods for identification of studies

#### Electronic searches

Unpublished and/or non-English-language manuscripts were considered for review inclusion. A comprehensive search of electronic databases (e.g., MEDLINE, EMBASE, SCOPUS, BIOSIS Previews and the Cochrane Library) using broad search terms was completed.

#### Searching other resources

The bibliographies of all included articles were examined to identify additional potentially relevant publications. Grey Literature including conference abstracts, websites and thesis were searched. This included Conference Papers Index and OCLC Papers First. Ongoing trials were identified using controlled trial registration websites, including ICRTP Search Portal for the World Health Organization. Attempts were made to contact the authors of unpublished materials or abstracts for additional data and material as needed. The authors of a recent narrative review were also contacted.

### Data collection and analysis

#### Selection of studies

All CCTs involving PVI solution in adult colorectal surgery were included. A trained librarian conducted the electronic searches, and one author conducted a pre-screen to identify the articles clearly irrelevant by title, abstract and keywords of publication. Two independent reviewers then assessed the remaining studies for relevance, inclusion, and methodological quality. Articles were classified as either: (1) Relevant (meeting all specified inclusion criteria); (2) Possibly relevant (meeting some but not all inclusion criteria); (3) Rejected (not relevant to the review). Two reviewers independently reviewed full text versions of all studies classified as relevant or possibly relevant. Disagreements were resolved by re-extraction, or third party adjudication, when necessary.

#### Data extraction and management

Two reviewers independently extracted data from the full versions of the manuscripts. The extracted information included details of methods (e.g., randomization, blinding, etc.), demographics (e.g., age, sex, etc.), clinical characteristics of each group, study inclusion and exclusion criteria, number of patients excluded and lost to follow up, details of intervention (e.g., strength, time prior to surgery, etc.), baseline and post-intervention outcomes (e.g., wound infections, abscess, transfusions, hospital LOS, re-hospitalization, dehiscence, re-exploration, sepsis, death, any health economic outcomes, etc.) and methods of analysis. Disagreements were resolved by re-extraction, or third party adjudication, when necessary. Records were maintained in order to complete the “Quality of reporting meta-analysis”.

#### Assessment of risk of bias in included studies

All included trials were assessed independently by two reviewers for methodological quality using the Cochrane (concealment of allocation) and Risk of Bias (RoB) tools [10]. Disagreements were resolved by re-extraction, or third party adjudication, when necessary.

#### Dealing with missing data

Attempts were made to contact the authors of included clinical trials to secure any missing information or data. Imputation of missing values was completed when appropriate.

#### Assessment of heterogeneity

Heterogeneity was assessed using X^2^ and I^2^ statistics. I^2^ heterogeneity cut points for heterogeneity were as follows [10]: (1) > 25%: low; (2) > 50%: moderate; (3) > 75%: high.

#### Assessment of reporting biases

##### 1) Data synthesis

Two independent reviewers completed data extraction; analysis was descriptive. Where possible and appropriate, dichotomous outcomes were reported and pooled as relative risk (RR) or odds ratio (OR) with their associated 95% confidence intervals (CI). Where possible and appropriate, continuous outcomes were reported as mean differences (MD) with associated 95% CIs and pooled as weighed (WMD) or standardized (SMD) MDs with associated 95% CIs.

##### 2) Subgroup analysis and investigation of heterogeneity

Planned subgroup analyses included: classifying the trials by use of preoperative antibiotics, age of the patients, and duration of follow-up.

##### 3) Sensitivity analysis

Sensitivity analyses were performed to explore the influence of factors such as quality of included studies, random effects vs. fixed effects model and by year of publication.

## Results

### Description of studies

#### Results of the search

**Figure 1:**
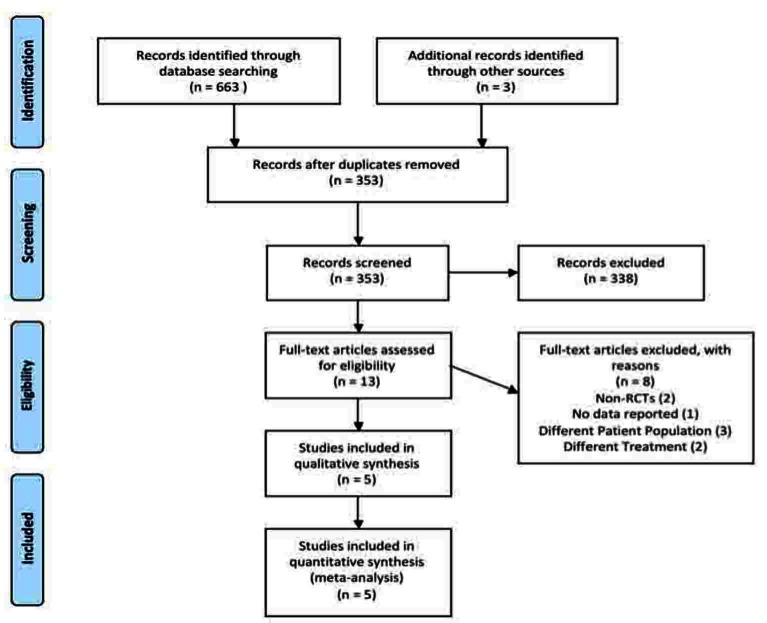
Systematic review PRISMA flow diagram.

A total of 353 articles were identified using our search criteria (Fig. 1). From this total, 10 CCTs meeting the inclusion criteria were identified following careful screening. Three articles were discovered after scanning the references of potential articles [11-13]. Thus, a total of 13 articles were assessed via examination of the full text using our inclusion/exclusion criteria. Ultimately 5 articles were included and all 5 articles were utilized for the meta-analysis.

#### Included studies

A total of 5 articles assessing PVI irrigation of subcutaneous tissues following colorectal operations were identified. The studies compared PVI irrigation or spray of subcutaneous tissues following fascial closure to a control group. Two of the studies used PVI-based spray [14, 15], while the 3 others used variations of PVI solution [12, 13, 16]. The control groups either received no treatment [12, 14, 15] or irrigation of subcutaneous tissues with normal saline [13] or iodine tincture to skin edges [16]. Detailed descriptions of the included studies are provided in Table 1.

**Table 1:** Description of Studies Included in Systematic Review

Author, Year of Study	Design, Duration	Treatment	Control	Participants						Outcomes
				PVI			Controls			
				+	Total	%	+	Total	%	
Gray, 1981	RCT, 15 months	PVI spray	Not sprayed	4	32	12	14	41	34	Incidence of wound infection
Kothuis, 1981	RCT, ?	10% PVI solution	Iodine tincture	10	19	47	6	11	45	Wound healing
McCluskey, 1976	RCT, ?	10% PVI solution	No solution	2	4	50	3	6	50	Incidence of infected wounds
Rogers, 1983	RCT, 6 months	10% PVI solution	Normal saline	1	24	4.2	5	27	18.5	Wound infection rate
Walsh, 1981	RCT, 12 months	5% PVI spray	Not sprayed	3	22	14	8	19	42	(1) Incidence of wound infection (2) Mean postoperative hospital stay
Totals	5 RCTs	3 solution; 2 spray	3 non-Rx	17	101	-	36	104	-	Variable

Note: RCT = Randomized Control Trial; + = Number of wound infections; PVI = Povidone-Iodine

Of the total number of patients included in all 5 studies, only a small percentage of patients were included in the meta-analysis. The baseline characteristics of the patients in the included studies are provided in Table 2. The mean age was between 40 - 60 years of age; however, it was unclear which characteristics corresponded to the patients included in the meta-analysis. Thus, subgroup analysis could not be preformed.

**Table 2:** Baseline Characteristics of Patients in Included Studies

Author, Year of Study	Total number of patients	# of patients included in SR	Age (years)	Gender		Operative time (min)	Preoperative antibiotics (# of patients)	Postoperative antibiotics (# of patients)
				M	F			
Gray, 1981	153	73	16 - 76	69	84	20 - 105	44	36
Kothuis, 1981	220	30	48 (mean)	132	88	< 60 - 240		
McCluskey, 1976	110	10	40 - 65	46	64	N/A		
Rogers, 1983	187	51	60.2 (mean)					
Walsh, 1981	627	41	43.4 (mean)	314	313		139	83

Note: SR = Systematic Review; M = Males; F = Females; min = minutes; N/A = not available

Wound infection after surgery was the primary outcome in all 5 RCTs. LOS, a secondary outcome measure, was only assessed by one RCT [15]. In this study, the authors reported an average decrease in post-operative stay of 5.3 days in patients receiving PVI compared to controls. It was not possible to investigate the LOS results in detail, since the data was not reported in an interpretable manner in the sole study providing this evidence. Mortality, sepsis, etc. were not reported in sufficient detail to be included in this review.

**Figure 2:**
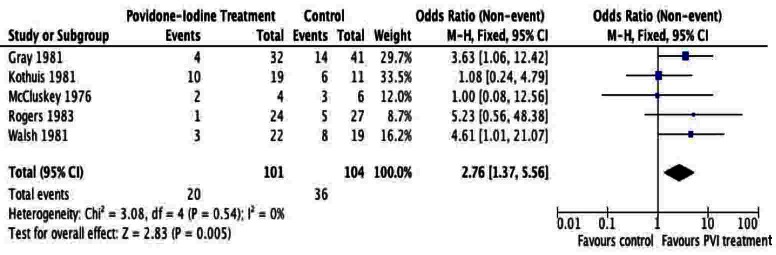
Forest plot of odds ratio for Povidone-Iodine treatment compared to controls.

**Figure 3:**
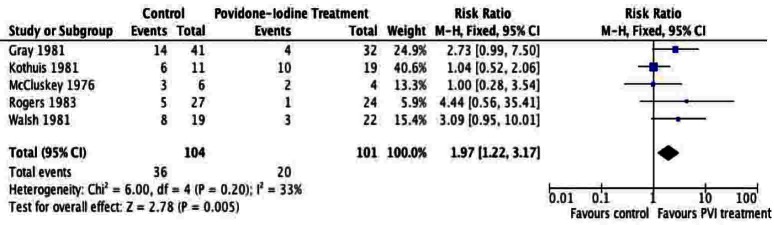
Forest plot of risk ratio for Povidone-Iodine treatment compared to controls.

A total of 205 patients were included from the included studies and were used for meta-analysis of the primary outcome. The meta-analysis of surgical site infection (Fig. 2, 3), demonstrated a reduction in surgical site infection for patients treated with PVI solution or spray for OR (2.76; 95% CI: 1.37 to 5.56) and RR (1.97; 95% CI: 1.22 to 3.17) analyses compared to controls for subcutaneous tissues prior to skin closure for elective colorectal operations. The heterogeneity of the included studies was low for both OR (I^2^ = 0%) and RR (I^2^ = 33%).

#### Excluded studies

A total of 8 articles were excluded based on specified criteria. The first article was removed because it was a narrative review on PVI in colorectal operations [6]. A second article was excluded due to lack of a control group, thus not meeting our criteria for a RCT [17]. Three articles were excluded for enrolling both elective and emergent abdominal operations, for which the data could not be differentiated [1, 11, 18]. Harihara et al used PVI topically on the skin rather than the subcutaneous tissue [19]. One article investigated multiple different treatments other than PVI irrigation [20] while another article was excluded for having no outcome data to report [21].

### Risk of bias in included studies

The Risk of Bias tool by Cochrane was used to assess bias in the 5 included studies [10].

#### Allocation

**Figure 4:**
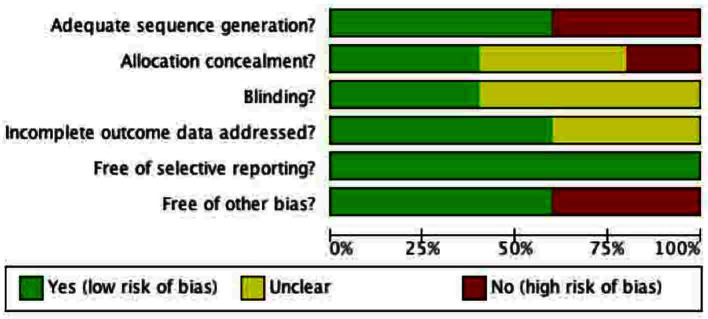
Methodological quality of included studies graph. Methodological quality graph: review authors’ judgments about each methodological quality item presented as percentages across all included studies.

**Figure 5:**
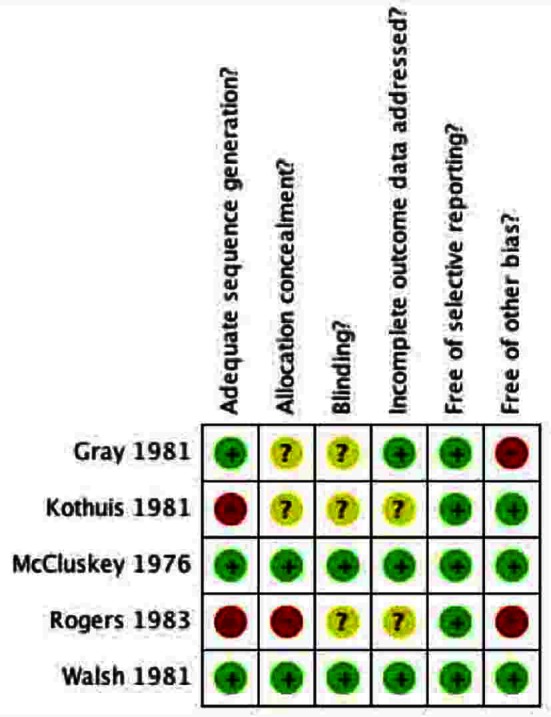
Methodological quality summary of included studies. Methodological quality summary: review authors’ judgments about each methodological quality item for each included study.

Allocation concealment was appropriately conducted by two of the five included studies (Fig. 4, 5). In both studies [12, 15] the surgeon was made aware of assignment of the patient following closure of the peritoneum. The other 3 studies provided no clear description of the concealment from the treating surgeon [13, 14, 16].

#### Blinding

The patients were unaware of their group assignment in all included studies. For example, the patients were adequately blinded in all studies because they were anesthetized during the surgical operation and were not made aware post-operatively. The surgeons could not be blinded in any of the studies because they either irrigated the subcutaneous tissues with PVI solution/spray or not. For unbiased reporting, the most important person to be blinded in these surgical studies was the outcome assessor, the person assessing the surgical sites for signs of infection. Two of the studies clearly stated that the outcome assessor was blinded to the group assignments and was independent of the surgical team [12, 15]. In two of the studies, no information was given regarding the person assessing for wound infections [13, 16]. In one study the surgical site was assessed by the house surgeon, however it was unclear if the house surgeon was involved in the operation [14].

#### Incomplete outcome data

Three of the studies adequately accounted for all patients in their trials [12, 14, 15]. This included accounting for excluded patients, for which justification was given. In the other two studies, no information was given about excluded patients [13, 16]. In Walsh et al, data was complete in assessing wound infection, however in terms of the secondary outcome, length of hospital stay, data was missing and not clearly explained [15].

#### Selective reporting

All of the five studies reported the primary outcome as wound infection. They all appear to be free of selective reporting with respect to the primary outcome.

#### Other potential sources of bias

Three of the studies appear to be free of other sources of bias [12, 15, 16]. Gray et al involved only a single surgeon, which could potentially bias the results [14]. The study by Rogers et al was conducted at a veteran’s hospital, which may be a source of bias because of the relatively older patient population [13].

## Discussion

### Summary of main results

Irrigation of subcutaneous tissues following elective colorectal operations is typically based on surgeon preference. This preference is perhaps based on training and clinical experience, and surgeons either support or refute the benefits of this management. Randomized controlled trials have assessed the use of PVI to irrigate subcutaneous tissues following abdominal operations; however, no randomized trials have specifically and solely assessed patients undergoing elective colorectal operations. The aim of this review was to systematically compare PVI irrigation to a control group in patients undergoing elective colorectal operations.

Using comprehensive search strategies to reduce publication bias and multiple reviewers to reduce selection bias, this review analyzed the available literature on the effects of PVI irrigation on surgical site infections. As seen in Figure 1, a total of 353 records were screened by title and abstract to render 13 eligible articles; five articles involving 205 patients were included in the systematic review. All five studies included in the review reported wound infection as the primary outcome, which enabled a meta-analysis of surgical site infection to be completed [12-16]. PVI subcutaneous tissue irrigation significantly decreased the risk of developing a surgical site infection following an elective colorectal operation. Insufficient data were available for robust corroborating evidence from any of the proposed secondary outcomes.

The 5 studies included in the review, were heterogeneous in the types of operation included; no study solely assessed elective colorectal operations. Therefore, only 5 - 45% of patients could be included in the review from each study. The demographics of the included patients could not be extracted to represent only the patients undergoing elective colorectal operations. Therefore age and sex of the patients could also not be elicited. Influencing factors such as pre-operative and post-operative antibiotics were provided in only two of the studies [14, 15], however it was unclear whether these patients were in the treatment or control groups. The protocol for the amount and method of PVI irrigation was heterogeneous among the included five studies. Three used PVI solution [12, 13, 16], in different quantities and concentrations, two used PVI spray for subcutaneous tissue irrigation [14, 15]. The control group in each of the studies also differed in terms of use of no irrigation or saline irrigation. One study used iodine tincture on the subcutaneous tissues on the wound [16].

### Overall completeness and applicability of evidence

In this systematic review, irrigation of subcutaneous tissues with PVI following elective colorectal operation reduced the risk of developing a wound infection. We critically appraised RCTs, which used any form of PVI irrigation in elective abdominal operations. Our search strategies were broad to maximize the potential to find relevant articles. This allowed the review to identify RCTs which may be heterogeneous in their patient population, but for which data would be relevant and potentially extractable. The exhaustive and comprehensive search strategies combined with an experienced librarian allowed our review to be thorough and complete.

The primary outcome, surgical site infection, was assessed by all the included studies in the review. The evidence revealed a significant benefit of PVI irrigation of subcutaneous tissues for patients undergoing elective colorectal operations. These results are based on the best evidence available; however, the review is limited by the small number of RCTs available. Moreover, the small number of patients (205 in total) included in the meta-analysis make the interpretation of the results difficult. Also, the lack of clear baseline characteristics of the patients included reveals a potential source of bias. It is possible that the patients in the treatment and control groups in these RCTs were not similar prior to the surgery. Also, patient factors known to influence post-operative infections such as diabetes, renal impairment and corticosteroid use were not taken into consideration.

### Potential biases in the review process

Systematic reviews are the best method of summarizing the available evidence; however, the quality of such reviews depends on the methods employed and the quality of the primary studies.

Evidence suggests that publication bias is pervasive in the literature [10]; however, negative trials are less likely to be published and more likely to be excluded from a review of this nature, potentially biasing the study conclusions. We believe that our comprehensive search strategy minimized such bias. We failed to identify unpublished trials; however, two trials included in this review were reported as ‘negative’ trials [12, 16]. Finally, we did not test the detection of publication bias through a funnel plot because they are subjectivity linked to their interpretation when the number of studies included is small.

Selection bias is another major concern in systematic reviews; however, we used an a priori protocol, multiple reviewers and an adjudication process when needed.

The quality of the primary studies was also a concern. The included studies used different randomization processes, which may be a source of bias because adequate randomization generates balanced groups to minimize the effect of known and unknown confounding variables. Only 3 studies [12, 14, 15] provided adequate descriptions of the randomization process. Furthermore, allocation of concealment was only described by two studies [12, 15]. Blinding of the outcome assessor prevents bias related to the primary outcome: wound infection. However this was only described clearly by two studies [12, 15] and thus may be a possible contributor to performance bias.

Some potential sources of heterogeneity (e.g., age, sex, duration of surgery, etc.) could not be addressed in this review due to the lack of consistent outcome reporting. Other possible sources of heterogeneity (e.g., study design, population and interventions) were sufficiently similar to support the decision to pool data.

Outcome reporting was variable and incomplete. For example, the definition of surgical site infection varied among the five studies. Combined with the relatively small numbers of included patients in each study, this is a potential source of bias. Only three of the studies provided information of patients lost to follow-up or excluded patients (attrition bias), which makes it difficult to decide if the study population is representative (external validity) [12, 14, 15].

**Figure 6:**
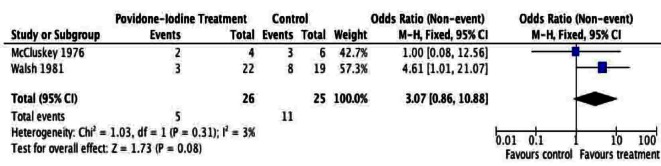
Sensitivity analysis based on high methodological quality.

**Figure 7:**
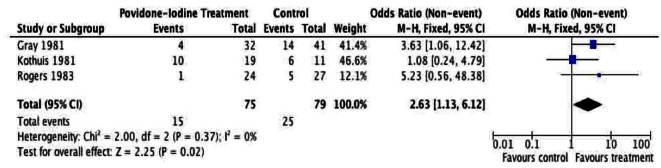
Sensitivity analysis based on low methodological quality.

Given the sources of bias, the results of our meta-analysis have to be cautiously interpreted. Small sample size, lacking demographic data, inexplicit randomization and allocation concealment are some of the most relevant factors that may produce misleading results. Five trials providing evidence addressing similar post-operative complications produced many examples of clinically relevant heterogeneity. The sensitivity analyses in Figures 6 and 7 demonstrate the effect on the odds ratio by the methodological quality of the RCTs. This calls for future efforts to standardize RCTs assessing surgical site infections. This systematic review and meta-analysis of PVI irrigation of subcutaneous tissues in patients undergoing elective colorectal operations could serve as an ideal basis for development of high-level RCT evidence.

### Agreements and disagreements with other studies or reviews

This is the first true systematic review to specifically investigate the effectiveness of PVI irrigation of subcutaneous tissues prior to skin closure in elective colorectal operations for the reduction of surgical site infection. This systematic review provides evidence that irrigation with PVI may reduce wound infection following elective colorectal operations. This finding is in disagreement with a recent narrative review [6]. The narrative review states that there is no conclusive evidence to support PVI; however, the authors did not perform a meta-analysis. The evidence based on five RCTs supports that there may be a benefit to PVI irrigation of subcutaneous tissues in adult patients undergoing elective colorectal operations.

### Conclusions

#### Implications for practice

Irrigation of subcutaneous tissues prior to skin closure in elective colorectal operations with PVI is associated with fewer surgical site infections, as far as can be assessed with the currently existing randomized trial literature.

#### Implications for research

To demonstrate conclusively that PVI irrigation of subcutaneous tissues prior to skin closure in adult elective colorectal operations reduces surgical site infection, larger and rigorously designed studies are needed. These randomized controlled studies need to focus solely on adult colorectal operations with a standardized method of PVI irrigation and a clearly described and clinically relevant definition of surgical site infection and other secondary outcomes.
